# Why Trolley Problems Matter for the Ethics of Automated Vehicles

**DOI:** 10.1007/s11948-019-00096-1

**Published:** 2019-03-04

**Authors:** Geoff Keeling

**Affiliations:** Department of Philosophy, University of Bristol, Cotham House, Bristol, BS6 6JL UK

**Keywords:** Automated vehicles, Ethics of risk, Trolley problems, Ethics of harm

## Abstract

This paper argues against the view that trolley cases are of little or no relevance to the ethics of automated vehicles. Four arguments for this view are outlined and rejected: the Not Going to Happen Argument, the Moral Difference Argument, the Impossible Deliberation Argument and the Wrong Question Argument. In making clear where these arguments go wrong, a positive account is developed of how trolley cases can inform the ethics of automated vehicles.

## Introduction

Automated vehicles (AVs) are expected to encounter road-traffic scenarios which present moral dilemmas.^1^ There is a dispute about what *kind* of moral dilemmas AVs will encounter. What some people have in mind are dilemmas where harming at least one person is unavoidable; and a choice is required about how to distribute harms or risks of harms between multiple persons whose interests are in conflict (Goodall 2014; Gurney 2016; Lin 2016; Leben 2017; Keeling 2018a, b). What others have in mind are dilemmas that arise in normal driving. For example, a scenario in which the AV must decide how heavily to brake when approaching a crossing given uncertainty about whether a pedestrian will step into the road (Nyholm and Smids 2016; Himmelreich 2018; Thornton 2018). On either view, there is a substantive moral question about which values ought to be reflected in the AV’s decision-making algorithm. Call this the *moral design problem*.^2^

One approach to the moral design problem involves asking what morality requires in *trolley cases*. In these cases: (1) the AV must choose one of two actions; (2) the AV knows what the consequences of each action will be; (3) each action imposes a distribution of benefits and burdens over at least two affected parties; and (4) the interests of these parties are jointly unsatisfiable. Jean-François Bonnefon and colleagues discuss one example in which the AV can continue its present course and cause the deaths of five pedestrians; or swerve to avoid the pedestrians but cause its passenger’s death in the process (Bonnefon et al. 2016).

The relevance of trolley cases to the moral design problem has been challenged in recent papers (Goodall 2016; Nyholm and Smids 2016; Himmelreich 2018). There are four serious charges: (1) trolley cases are not going to happen in the real-world; (2) there is a substantive moral difference between trolley cases and real-world collisions, e.g. real-world collisions involve decision-making under risk and uncertainty and trolley cases do not; (3) it is impossible for AVs to deliberate in accordance with answers to the moral design problem based on trolley cases, as these cases presuppose a ‘top-down’ approach to AV engineering; and (4) trolley cases provide a moral answer to what is ultimately a political problem, in the sense that the values encoded into AV decision-making algorithms ought to reflect a compromise between the values of the relevant stakeholders. This paper defends trolley cases against these four charges, and in doing so, develops a positive account of how trolley cases might inform the ethics of AVs.

## The Not Going to Happen Argument

According to what might be called the *Not Going to Happen Argument*,

P1: Trolley cases are relevant to what morality requires in dilemmas where harming at least one person is unavoidable, and a choice is required about how to distribute harms between multiple persons whose interests are in conflict.
P2: AVs will not encounter dilemmas like these.
C: Therefore, trolley cases are irrelevant to the moral design problem.

This argument is unsound. The standard response holds that P2 is false, i.e. that there are plausible examples of trolley cases which AVs might encounter in the real-world (Goodall 2014: 60–61; Lin 2016: 71–72; Santoni de Sio 2017: 416–417; Keeling 2018a: 425). In one of these examples, the AV is travelling on a two-lane bridge. A bus in the other lane swerves into the AV’s lane. The AV can either brake, in which case it will collide with the bus; or it can swerve into the other lane, in which case it will hit the side of the bridge (c.f. Goodall 2014: 60). Cases like these seem plausible. But in recent work the philosopher Johannes Himmelreich (2018: 673–674) has argued that AVs could not be in trolley-style scenarios whilst at the same time maintaining sufficient control to make a decision. Roughly speaking, Himmelreich’s point is that a trolley-style dilemma is unlikely to arise if the AV is travelling sufficiently slowly to make a meaningful decision; and that in the sorts of high-speed cases where a trolley-style dilemma might arise, it is unlikely that the AV would have sufficient control to make a decide how to allocate benefits and burdens between the affected parties. So, grant P2 for the sake of argument. It is argued that the Not Going to Happen Argument is unsound conditional on the assumption that AVs will not encounter trolley cases in practice.

First, the Not Going to Happen Argument is deductively invalid, in the sense that it is possible for P1 and P2 to be true and for C to be false. To *guarantee* the truth of C, a third premise is required:

P3: Cases of type X are relevant to the moral design problem only if AVs will encounter cases of type X in the real-world.

This premise is false. The mistake is most obvious when the Not Going to Happen Argument is applied to a law in physics. Consider the Ideal Gas Law,

$$PV = nRT$$

This law describes the relation between certain properties of gasses. It holds that the product of the pressure and volume of a gas is equal to the amount of gas in moles multiplied by the gas constant multiplied by the absolute temperature. This law applies to *ideal* gasses. An ideal gas consists of many randomly moving particles with no spatial extension engaging in perfectly elastic collisions. There are no ideal gasses. But it would be a mistake to conclude that the Ideal Gas Law is irrelevant to the behaviour of real-world gasses. This is because in a broad class of cases the Ideal Gas Law accurately describes how changes in some of the properties of a gas make a difference to other properties of the gas.

The same point can apply to trolley cases. These cases presuppose a model of morality according to which some acts are morally permissible, others are morally required, and others are morally impermissible.^3^ Some examples: (1) it is morally permissible to help old people across the street; (2) it is morally required to save someone from severe harm if doing so imposes only a small personal cost; and (3) it is morally impermissible to torture babies. First, ‘morally permissible’, ‘morally required’ and ‘morally impermissible’ are properties of acts, in the sense that it is true or false of a given act that it is morally permissible, morally required or morally impermissible. Second, the moral permissibility of an act *depends on* certain other properties of that act. These are called *morally relevant properties*. Presumably, what makes it the case that torturing babies is morally impermissible is that this act has the property of causing gratuitous harm. So, causing gratuitous harm is morally relevant. Third, the point of asking what morality requires in trolley cases is to determine under idealised conditions how some properties of acts make a difference to the moral permissibility of those acts (Kagan 1988: 5–8, 2016: 151–154; Kamm 2016: 13). The not unreasonable hope is that the relations between the properties of acts and their moral permissibility in *ideal* cases is relevant to the moral permissibility of acts which instantiate those same properties in more noisy real-world cases.^4^

This is all quite abstract. Keeping in mind the model of morality described above, here are two practical examples of how trolley cases might be used to decide between competing answers to the moral design problem. First, in a recent paper, Julian de Freitas, Sam E. Anthony and George A. Alvarez write that:

The main safety goal for any driver – human or machine – is to avoid harm […] Unfortunately, both humans and today’s best computer systems are imperfect at it. Even so, the substantial improvements that we rightfully expect from future AV systems are utterly unlikely to come from considering trolley dilemmas. (de Freitas et al. 2019: 4)

It is true that trolley cases will not help to build AV systems that are better at avoiding harm than existing AV systems. But this is like criticising a hammer for being a bad screwdriver. Trolley cases are not a computer programming tool, so it is reasonably obvious that trolley cases will have no useful application in this domain. However, trolley cases are relevant to the claim that avoiding harm ought to be the main safety goal for AVs. This claim, interpreted in the terminology of the model sketched above, holds that the only property which makes a difference to the moral permissibility of the AV’s acts is the property of causing harm. Trolley cases provide good reason to believe that this claim is false. Consider the philosopher Foot’s (2002) original

*Trolley Driver:* A trolley’s brakes fail. The driver can continue on the same track and kill five workmen; or steer the trolley onto another track, saving the five workmen but killing one workman on the other track.

And

*Transplant:* A surgeon can kill one patient and use their organs to save the lives of five other patients.

If Freitas, Anthony and Alvarez are correct that harm is all that matters, then the trolley driver is morally required to kill one person to save five; and the surgeon is morally required to kill one patient and harvest their organs to save five others. In both cases, these actions would cause the least harm. But whilst most people have the intuition that it is permissible to redirect the trolley in Trolley Driver, most people also have the intuition that it is morally impermissible to kill one to save five in Transplant. It would be a serious moral error if the surgeon killed one patient with the express intention of harvesting their organs to save five others. Presumably, what explains the difference in intuitions here is that the cases differ in some morally significant respect. In other words, there is a property other than harm contributing to the moral permissibility of the actions. But if this is true, then Freitas, Anthony and Alvarez are incorrect that harm is the *only* property of actions which makes a difference to moral permissibility. So, one application of trolley cases to the moral design problem is that trolley cases can be used to argue against theories of which properties make a difference to moral permissibility.

It might be objected that Trolley Driver and Transplant are too divorced from the context of AV collisions for the relations between the properties of *these* acts and moral permissibility to be relevant to the moral design problem. But it is possible to make trolley cases which are closer to the context of AV collisions. For example, suppose that a motorcyclist is skidding across the road towards a crowd of pedestrians on the pavement. The AV can brake, in which case the motorcyclist will skid into the pedestrians and cause their deaths. The AV could also accelerate into the motorcyclist, in which case the motorcyclist would be killed, but the skid would be deflected and the pedestrians would be unharmed. Presumably, it is morally permissible for the AV to brake here. It is too demanding to suppose that the AV is morally required to intervene and kill the motorcyclist. But if Freitas, Anthony and Alvarez are correct that harm is all that matters, then the AV is morally required to kill the motorcyclist. The salient point here is that properties other than harm seem to make a difference to the moral permissibility of the AV’s acts in ideal cases. It seems that whether or not the harm is *done* or *merely allowed to happen* is morally relevant. Presumably, the properties of doing harm and allowing harm to happen are instantiated by some acts available to AVs in the real-world. So, absent good reason to believe that the relation between these properties and moral permissibility is *radically different* in real-world cases, the view that avoiding harm is all that matters in AV collisions ought to be discarded.

This first illustration of the relevance of trolley cases to the moral design problem was negative: trolley cases can be used to show that theories of what matters morally in AV collisions are false. In making precise which properties of acts make a difference to moral permissibility in cases like Trolley Driver and Transplant, it is also possible to formulate positive arguments about which properties AVs should take into account when making decisions. Here is an example: Foot’s explanation of the conflicting intuitions in Trolley Driver and Transplant appeals to *positive* and *negative* duties. Foot argues that Trolley Driver involves a conflict between two negative duties: the duty not to kill one and the duty not to kill five. The duty not to kill five is stronger than the duty not to kill one, so it is morally permissible to kill one to save five others. In contrast, the conflict in Transplant is between the negative duty not to kill one and the positive duty to aid five others. It is better, on Foot’s view, to let five people die than it is to kill one person (Kamm 2016: 15–16; Thomson 2008: 360, 2016: 114). So, Foot’s explanation of why it is permissible to kill one to save five in Trolley Driver but not in Transplant is that Trolley Driver is a choice between *killing one* and *killing many*, whereas Transplant is a choice between *killing one* and *letting many die*. Suppose Foot is correct that this distinction is the best explanation of the conflict in intuitions. Then there is good reason to ensure that AVs are sensitive to the moral difference between killing and letting die.

To conclude: The Not Going to Happen Argument holds that trolley cases are irrelevant to the moral design problem *because* AVs will not encounter trolley cases in the real-world. This argument falsely assumes that trolley cases are relevant to the moral design problem only if AVs will encounter these cases in practice. What matters for the relevance of these cases to practical ethical dilemmas is that the acts in practical dilemmas instantiate some of the morally relevant properties which trolley cases are concerned with. It is not unreasonable to suppose, for example, that whether an AV kills someone or merely allows them to die makes a difference to the moral permissibility of the AV’s actions. So, trolley cases are relevant to the moral design problem *even if* AVs will never encounter trolley cases in practice.

## The Moral Difference Argument

According to the *Moral Difference Argument*, trolley cases and real-world collisions are different in at least some morally significant respects; and these differences render trolley cases of little or no relevance to the moral design problem.

The simplest version of this argument holds that trolley cases ignore at least some properties of the AV’s acts which make a difference to the moral permissibility of those acts in real-world collisions (c.f. Nyholm and Smids 2016). Two examples: (1) Trolley cases exclude information about which of the affected parties is responsible for the collision. In real-world collisions, facts about who is responsible in part explain the moral permissibility of the AV’s acts. (2) Trolley cases omit information about special obligations. In real-world collisions, it might be that the AV manufacturer has an obligation to protect the welfare of the AV’s passengers and that this contributes to the moral permissibility of the AV’s acts.

The insight here is that trolley cases cannot provide the *entire* answer to the moral design problem. This is unsurprising. It is almost never true that a set of imagined cases provides sufficient reason to accept a view about morality. But this does nothing to diminish the relevance of trolley cases to the moral design problem. First, suppose that trolley cases are used to argue that a property such as *letting people die* in part contributes to the moral permissibility of the AV’s acts. The fact that there exist other morally relevant properties in real-world collisions does not render the property *letting people die* less relevant than it otherwise would be. It is either morally relevant or it is not. Second, suppose that a trolley case is used as a counterexample to an account of the morally relevant properties in collisions. If the account holds that one property, e.g. harm, is morally relevant, then the existence of *other* morally relevant properties cannot be invoked to diminish the relevance of the trolley case. The existence of other morally relevant properties implies the falsity of the account being defended. If the account holds that multiple properties matter morally, then for analogous reasons additional morally relevant properties cannot be invoked to diminish the relevance of a trolley case employed as a counterexample to that account. These are the only two obvious uses for trolley cases in the dispute over the moral design problem. So, it does not matter for the relevance of trolley cases that there exist other morally relevant properties.

A better version of the Moral Difference Argument holds that the moral considerations in trolley cases are *categorically* different to the moral considerations in real-world collisions. So, trolley cases are of little or no relevance to the moral design problem. The first step in this argument is to establish a non-normative difference between trolley cases and real-world collisions. Standardly, it is argued that in trolley cases the AV knows the outcome of the collision conditional on each action. But in real-world collisions one action might produce several different outcomes, and the AV at best has a probability distribution over these outcomes. In short, AV collisions involve *risk* (Himmelreich 2018: 676–677; Nyholm and Smids 2016: 1286). The second step holds that the non-normative difference between these cases gives rise to a normative difference. The claim here is that the *presence* of risk in collisions renders the moral dilemmas in these cases different in kind to those in trolley cases. The philosophers Sven Nyholm and Jilles Smids write that:

Reasoning about risks and uncertainty is categorically different from reasoning about known facts and certain outcomes. The key concepts used differ drastically in what inferences they warrant. And what we pick out using these concepts are things within different metaphysical categories, with different modal status (e.g. risks of harm, on one side, versus actual harms, on the other). (Nyholm and Smids 2016: 1286)

Once the distinction between risky and non-risky cases is made precise, it is clear that the categorical difference described here is insufficient to render trolley cases of little or no relevance to the moral design problem.

Take the orthodox model of decision-making under risk (Luce and Raiffa 1957; Savage 1972). In this model a risky decision involves a set of mutually-exclusive and exhaustive possible worlds, $\omega_{1} , \ldots ,\omega_{n} \in {\mathcal{W}}$; and the AV is uncertain about which of these worlds is the actual world. In a simple case, the AV might be uncertain about whether or not a pedestrian will step out into the road. This scenario can be modelled with two worlds: one in which the pedestrian steps out into the road, and the other where the pedestrian does not.

The AV must choose one action from a set ${\mathcal{A}}$ containing at least two alternatives. These might be swerving or continuing its present course. The outcome of the decision is determined by both the AV’s action and which world is the actual world, i.e. the outcome of the same action is different in different worlds. Formally, let $\chi$ be the set of outcomes. Each $x \in \chi$ might roughly be understood as the *consequences* of performing an action in a particular world. But more precisely, these outcomes are to be understood in the broadest possible sense, so as to include facts about certain kinds of actions having been performed (Broome 1991: 4–5; Scheffler 1982: 1–2). Each action $a \in {\mathcal{A}}$ is a function, $a{:}{\mathcal{W}} \to \chi$, that maps each world $\omega_{i}$ to the outcome $x_{i}$ of performing $a$ in $\omega_{i}$. Thus, in this model, if two acts have the same outcomes across all possible worlds, these acts are equivalent.

The AV has a utility function, $u{:}\chi \to {\mathbb{R}}$, which assigns to each outcome $x \in \chi$ a cardinal representation of the moral value of that outcome. Given the broad definition of *outcome*, the utility function can incorporate deontic considerations such as the moral value of an outcome where the pedestrian is *killed* rather than *allowed to die*. The AV also has a credence function, $cr{:}{\mathcal{F}} \to \left[ {0,\;1} \right]$, which takes as inputs sets of worlds in a finite algebra ${\mathcal{F}}$ over ${\mathcal{W}}$, and outputs the AV’s degree of belief or credence in the proposition that each set of worlds contains the actual world.^5^ The acts in ${\mathcal{A}}$ are then evaluated from this position of uncertainty using a decision-rule. The best-known decision-rule is *expected utility maximisation*, according to which the AV should select the action which maximises the weighted sum of utilities over the different possible worlds, where the weights are determined by the AV’s credence in each world being the actual world. In formal terms, the AV should select the act which satisfies:

$$\mathop {\arg \hbox{max} }\limits_{{a \in {\mathcal{A}}}} \mathop \sum \limits_{i = 1}^{n} cr\left( {\omega_{i} } \right)u\left( {x_{i} } \right)$$

The difference that Nyholm and Smids identified between risky and non-risky cases can now be stated precisely. In non-risky cases, the AV knows which world $\omega_{i} \in {\mathcal{W}}$ is the actual world. So, each act can be evaluated using the outcome that it maps to in the actual world. In risky cases, it is necessary to consider different possible worlds when evaluating acts, as the moral value of an act depends on the value of the outcomes in different possible worlds. This difference is insufficient to render trolley cases irrelevant to the moral design problem. This is because trolley cases are relevant *only* to the AV’s utility function, in the sense that these cases are used to identify which properties are relevant to the evaluation of actions (e.g. harm, responsibility, fairness, and so on). Though risky cases present an additional problem of how to reason about utilities across worlds, the question of which properties the AV’s utility function should track is the same in risky and non-risky cases. In a sentence: there is no point talking about *expected* utility if the question of *what utility is* has not been settled (Crisp 2006: 39–40). So, the categorical moral difference between trolley cases and real-world collisions is not sufficient to render trolley cases of little or no relevance to the moral design problem.

## The Impossible Deliberation Argument

According to the *Impossible Deliberation Argument*, AVs cannot deliberate in accordance with answers to the moral design problem based on trolley cases. This is because answers based on trolley cases presuppose a ‘top-down’ approach to AV decision-making (Gurney 2016: 208; Himmelreich 2018: 675; Nyholm 2018: 5). What it means for an AV to be programmed in a top-down fashion is that the AV follows a set of rules in collisions, where these rules are expressible in the language of first-order logic (Alaieri and Vellino 2016: 161–162; Allen et al. 2005: 149–151). The top-down approach contrasts with a ‘bottom-up’ approach, on which the AV’s actions are determined by a connectionist algorithm with no explicit rules. The problem is that AV decision-making algorithms standardly use a bottom-up approach, so it is unclear how answers to the moral design problem based on trolley cases will be implemented into AVs. Himmelreich writes that:

[trolley] cases naturally lend themselves to a top-down design approach. But given the current prominence of the bottom-up approach in artificial intelligence in the form of neural networks, there is a risk of a discontinuity of approaches between ethics and engineering. (Himmelreich 2018: 675)

This argument is problematic for those who use trolley cases as inputs to so-called *collision optimisation algorithms*. These algorithms are sets of instructions for how AVs should behave in trolley-style collisions. More precisely, they are functions which take trolley cases as inputs and return a set of acts which the AV is morally permitted to perform in each case. Collision optimisation algorithms have been developed which reflect different answers to the moral design problem. The philosopher Leben (2017), for example, defends an algorithm on which the AV should compare the survival probabilities of the affected parties conditional on each act, and select the act which has the greatest minimum survival probability.

It is more or less clear that AVs cannot deliberate in accordance with algorithms like these. On one hand, these algorithms make implausible assumptions about the AV’s knowledge. There is no obvious reason to assume that the AV is certain about how many affected parties are in the collision; so, there is no obvious reason to assume that the AV will be certain about the survival probabilities of each party (Keeling et al. forthcoming). On the other hand, these algorithms require a top-down approach to AV decision making. Presumably, Himmelreich (2018: 675) is correct about the top-down approach being discontinuous with engineering practice. So, when trolley cases are used in *this* way, the Impossible Deliberation Argument presents a decisive objection.

But this is not the only way to use trolley cases. There is a distinction in utilitarian ethics between decision-procedures and criteria for rightness. A decision-procedure is a method of deliberation. A criterion of rightness is what explains the moral permissibility of actions (Brink 1986: 421; see also Bales 1971; Crisp 1992). A utilitarian might believe that what ultimately explains the moral permissibility of actions are facts about the degree to which those actions promote or maximise utility. But that does not commit them to the further view that people are morally required to deliberate in accordance with a utilitarian calculus. It might be better from the point of view of utility if people deliberated in accordance with certain moral virtues such as gratitude and beneficence (Hooker 2000; Crisp 1992).

There is a rough but important sense in which those designing collision optimisation algorithms are committed to the view that a good answer to the moral design problem is a decision-procedure for AVs, i.e. a set of instructions for how AVs should deliberate in the event of a collision. A more plausible view is that a good answer to the moral design problem is an account of the right-making properties of the AV’s actions in collisions. In other words, the answer explains *what matters* from the moral point of view, which might be utility, fairness, justice, rights, responsibility, justifiability to the affected parties, or some combination of these. The aim of such an account is to inform a process of value-sensitive design; that is, a process in which engineers, moral philosophers and other stakeholders work together to determine the ethical implications of technical decisions made in the design-process (Thornton 2018; van de Poel 2013). There is no need for philosophers to design collision optimisation algorithms in order to explain the considerations relevant to determining right-action in collisions. Presumably, if the right-making properties of the AV’s acts in collisions are made precise, these properties can inform a process of value-sensitive design.

This is all quite abstract. So, consider an example. The philosopher Kamm (2016: 62–67) argues that the Principle of Permissible Harm best captures our intuitions about what morality requires across a broad class of idealised cases. The philosopher Shelly Kagan describes the basic idea of this principle as follows:

Sometimes someone who is killed is killed by an event that is the very same event as saving a larger number of people (the greater good). Then the killing of one may be justified. But in other cases, the one who is killed is killed by something that is merely a casual *means* to the event that is the saving of the larger number. In such cases killing the one is not justified. So the crucial question is whether the event that results in the killing of the one literally constitutes the saving or is merely a means to that saving. (Kagan 2016: 156)

Here are two paradigmatic cases to illustrate the distinction that Kamm draws in the Principle of Permissible Harm. Consider,

*The Fat Man on Bridge Case:* George is standing on a bridge next to a fat man who is leaning over the barrier looking at the tracks below. An out of control trolley is heading towards five people. George can let the five people die, or he can push the fat man off the bridge, stopping the trolley but killing him in the process (Thomson 1976: 207–208).

If George pushes the fat man, then the fat man’s death would be a mere causal means to saving the five people on the track. The fat man being killed is what causes the five others to be saved. The Principle of Permissible Harm rules out pushing the fat man as it prohibits killing one *merely* as a causal means to saving five (Kamm 2016: 63). Contrast this with a paradigm case of a person being killed as part of the same event as saving five others:

*The Bystander Case:* An out of control trolley is heading towards five people. A bystander can pull a switch to divert the trolley onto another track, but then one person on the other track will be killed.

The Principle of Permissible Harm permits the bystander to divert the trolley, as the one person’s death makes no causal contribution to saving the five. The one person’s death is a causal consequence of the event that saves the five, namely the redirecting of the trolley. In this respect, the one person’s death is better understood as part of the same event as saving the five, as opposed to a causal means to that event.

This paper is not a defence of Kamm’s Principle of Permissible Harm. The point here is that Kamm’s principle is the sort of consideration which could inform a value-sensitive design process. The discussion on the trolley problem provides considerable insight into the properties which make a difference to the moral permissibility of acts. Kamm’s principle does a better job than most of explaining our intuitions about what is permissible across a broad class of cases. So, used like *this*, trolley cases are relevant to the moral design problem.

## The Wrong Question Argument

The final argument concedes that trolley cases are relevant to what *morality* requires in AV collisions. But it contends that the values which ought to be encoded into AV decision-making algorithms are not determined by moral considerations. Call this the *Wrong Question Argument*.

Himmelreich (2018: 675–676) argues that there is no unanimous agreement about which moral principles are true. So, any answer to the moral design problem based on trolley cases is unlikely to receive ‘broad societal acceptance’. This is because such an answer will appeal to principles which many people in society reject. Himmelreich believes that broad societal acceptance is a necessary condition for a successful solution to the moral design problem. So, what is required is not an answer to what *morality* requires in AV collisions, but instead an answer to a social choice problem. In Himmelreich’s words:

A trolley case prompts us to make an individual choice when what we in fact face is a social choice. What seems needed is a kind of compromise to overcome disagreements over issues of value. (Himmelreich 2018: 676)

Himmelreich is correct that broad societal acceptance is a necessary condition for a successful answer to the moral design problem. But it is unclear *why* a solution based on moral principles would not receive broad societal acceptance. Presumably, Himmelreich’s concern is legal moralism: the public would not accept a situation where AV manufacturers are legally required to encode certain moral values into AVs on the basis of *moral arguments*. But this is an exaggeration. Most people accept a modest form of legal moralism, according to which the moral wrongness of an action gives a *pro tanto* reason for criminalisation (cf. Duff 2014: 217). That is, a reason which ‘has genuine […] weight, but nonetheless may be outweighed by other considerations’ (Kagan 1989: 17; see also Broome 2013: 51–54). The strongest arguments for criminalising theft, non-fatal offences against the person and murder are to some extent reliant on moral considerations. Yet these offences receive broad societal acceptance. So, it is not unreasonable to suppose that moral arguments might be used in answering the moral design problem.

Furthermore, Himmelreich’s commitment that an answer to the moral design problem should receive broad societal acceptance is insufficient to establish the stronger claim that the moral design problem is a social choice problem. Social choice theorists are interested in ‘[whether] it is formally possible to construct a procedure for passing from a set of known individual tastes to a pattern of social decision-making, the procedure in question being required to satisfy certain natural conditions’ (Arrow 1963: 2). The standard way of making this question precise requires us to suppose that each individual in society has a preference ordering over a set of alternative policies. The challenge is then to describe a *social welfare function*, which takes the individual preference orderings as an input, and outputs a collective preference ordering over the policies. Social choice theorists dispute which formal properties the social welfare function should satisfy (*Ibid.*: 23; Sen 1970: 35–36). If the moral design problem is a social choice problem, then the values which ought to be encoded into AV algorithms *depend on* the preferences, tastes, or values of all the individuals in society. But the dependence relation is *functional*. What this means is that, taken together, the preferences, tastes, or values of each person in society *uniquely determine* the principles which ought to regulate AV behaviour.^6^

Himmelreich claims that broad societal acceptance is a necessary condition for a successful answer to the moral design problem. This might be true. But it does not follow that our problem is *essentially* one of aggregating individual tastes, preferences or values. This is true only if broad societal acceptance is both a necessary and sufficient condition for a successful answer to the problem. And there are reasons to accept or reject solutions to the moral design problem which do not pertain to social choice. On one hand, if a collective judgement holds that AVs should act in accordance with immoral principles, then there is a moral reason to reject that solution to the moral design problem. On the other hand, if there is a moral difference between, for example, killing and letting die, then there is a *pro tanto* reason for this distinction to be reflected in AV decision-making algorithms. This reason has genuine weight irrespective of whether the killing and letting die distinction is reflected in the values of society taken as a whole. Himmelreich’s best response is to accept that the moral design problem is not *essentially* a social choice problem; and to instead claim that the problem has a social choice element. This is correct. It does matter that the values put into AV driving algorithms receive broad societal acceptance. But this weaker view does not support the argument against trolley cases, as whilst trolley cases are not relevant to social choice problems, these cases are relevant to what is morally required in AV collisions.

## Conclusion

The confusion about trolley cases and their role in AV ethics is explained by two factors. On one hand, some philosophers have used trolley cases as inputs to so-called collision optimisation algorithms. These algorithms provide instructions for what AVs should do in the event of a trolley-style collision. This use of trolley cases invites the criticisms that trolley cases will not arise in practice; that there are substantial moral differences between trolley cases and the moral dilemmas in real-world collisions; and that answers to the moral design problem based on trolley cases make implausible assumptions about the AV’s knowledge and decision-making algorithm. On the other hand, critics of trolley cases have downplayed the fact that trolley cases have a broader application than their current use in the literature on AV ethics. Philosophers such as Kamm (1996, 2007, 2016) and Thomson (1976, 1990, 2008) have developed novel ethical principles which try to explain our moral intuitions across a broad class of idealised cases. Kamm’s Principle of Permissible Harm, for example, is *precisely* the sort of principle which deserves attention in the value-sensitive design process. Thus, there is a place for trolley cases in the ethics of AVs. But it is rather different from the place that philosophers have assigned it in the discussion so far.

## Footnotes

1 Here the term ‘automated vehicle’ (AV) refers to Level 5 automated vehicles in accordance with the Society for Automotive Engineers autonomous vehicle classification scheme. These vehicles require no human supervision or intervention in *any* circumstances.

2 Two points on the moral design problem. First, earlier formulations of the moral design problem concern *only* road-traffic scenarios where harm to at least one person is unavoidable; and a choice is required about how to distribute harms or risks of harm between multiple persons whose interests are in conflict (Keeling 2018a: 414, b: 259). The formulation in this paper is broader insofar as it also concerns the values relevant to determining AV behaviour in mundane traffic situations (c.f. Himmelreich 2018). Second, the moral design problem is not the only ethical question relevant to AVs. There are several other issues, such as identifying and minimising the risks associated with the transition to AVs; and how responsibility is distributed for harm or property damage caused by AVs. These issues require at least as much attention as the moral design problem. But the existence of these other issues does not entail or suggest that the moral design problem is unimportant. I am grateful to an anonymous reviewer for pressing me on this point.

3 The terms ‘morally right’ and ‘morally wrong’ could be used instead. But ‘morally right’ is ambiguous between morally permissible and morally required. So, these terms are used to avoid ambiguity. The properties relate as follows: an act is morally required just in case it is not permissible to not perform that act; and an act is morally impermissible if, and only if, that act is not morally permissible.

4 The philosopher Frances Kamm writes: ‘we can see why the cases that have been the focus of attention in trolley problem discussions seem artificial and unrealistic. They are specifically constructed, like scientific experiments, to distinguish among and test theories and principles […] because one theory or principle would imply the permissibility of conduct that the other theory or principle would deny. Using our intuitive judgements about which implications for cases are correct helps us decide among, and also revise, theories and principles’ (Kamm 2016: 13).

5 First, Sven Nyholm has said in personal correspondence that he thinks of these cases as involving objective probability and not subjective probability. The response to the Moral Difference Argument applies irrespective of whether objective or subjective probabilities are used. Second, the finite algebra ${\mathcal{F}}$ over ${\mathcal{W}}$. as the following properties: (1) ${\mathcal{W}} \in {\mathcal{F}}$ (2) $X \in {\mathcal{F}}$ implies that its complement $\left( {{\mathcal{W}} - X} \right) \in {\mathcal{F}}$ (3) if $X_1, X_2 \in {\mathcal{F}}$, then $X_{1} \cup X_{2} \in {\mathcal{F}}$. Third, the AV’s credence function is assumed to be a probability function.

6 It might be objected that I have interpreted Himmelreich’s use of the term *social choice* too narrowly. I have understood social choice in the way that Arrow (1963) and subsequent social choice theorists understood the term. But there is a pre-Arrow sense of social choice which is not concerned with spaces of aggregation functions, but instead with particular aggregation functions. This was the approach of the Marquis de Condorcet. Even so, on this broader meaning of social choice, the preferences, tastes or values of all the individuals in society *uniquely determine* the collective judgement. The point here is that independent moral considerations may still be relevant to the values which there is best reason to encode into AV driving algorithms. So, my point stands. I am grateful to Richard Pettigrew and Johannes Himmelreich for pressing me on this issue.
